# The German version of the tablet‐based UCSF Brain Health Assessment is sensitive to early symptoms of neurodegenerative disorders

**DOI:** 10.1002/brb3.3329

**Published:** 2023-12-02

**Authors:** Gianina Toller, Lorena Stäger, Dilaxy Kumurasamy, Patrick Callahan, Florian Köhn, Thomas Münzer, Ursi Kunze, Andreas U. Monsch, Kate Possin, Katherine P. Rankin, Ansgar Felbecker

**Affiliations:** ^1^ Department of Neurology Kantonsspital St. Gallen Gallen Switzerland; ^2^ Memory and Aging Center University of California San Francisco San Francisco California USA; ^3^ Geriatrische Klinik St. Gallen Gallen Switzerland; ^4^ Memory Clinic, University Department of Geriatric Medicine Felix Platter Basel Switzerland

**Keywords:** cognitive screening, major neurocognitive disorder, mild neurocognitive disorder, primary care, tablet‐based testing

## Abstract

**Introduction**: Cognition often remains unassessed in primary care. To improve early diagnosis of neurocognitive disorder (NCD) in Switzerland, the tablet‐based UCSF brain health assessment (BHA) and brain health survey (BHS) were validated.

**Methods**: The German BHA, BHS, and Montreal Cognitive Assessment (MoCA) were administered to 67 patients with mild/major NCD and 50 controls. BHA includes subtests of memory, executive, visuospatial, and language functioning, and informant‐based BHS asks about behavior and motor functioning.

**Results**: The complete instrument (BHA + BHS) was most accurate at detecting mild NCD (AUC = 0.95) and NCD without amyloid pathology (AUC = 0.96), followed by the BHA. All measures were accurate (all AUCs > 0.95) at distinguishing major NCD and NCD with amyloid pathology (Alzheimer's disease [AD]) from controls.

**Discussion**: The German BHA and BHS are more sensitive to mild NCD and non‐AD presentations than the MoCA and thus have a high potential to identify patients with NCD in primary care earlier than currently used screens.

## Graphical abstract

We show that a novel, tablet‐based assessement is sensitive to detect patients with neurodegenerative diseases as early as in the mild cognitive impairment stage. Our results show that the novel tool is superior to existing paper‐and‐pencil‐based instruments like the Montreal Cognitive Assessment (MoCA).

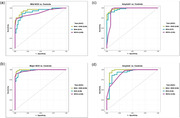

## 1 INTRODUCTION

Primary care providers (PCP) play a key role in the diagnostic process of neurocognitive disorders because they are often the first to evaluate patients presenting with progressive changes in cognition and behavior (Cardarelli et al., 2010), which are among the earliest clinical signs of neurodegenerative disorders (Ossenkoppele et al., 2015; Seeley et al., 2005). Despite the importance of early recognition and evaluation of cognitive and behavioral symptoms, neuropsychological measures that are sensitive to early symptoms of typical and atypical neurodegenerative diseases are not commonly part of the diagnostic workup in primary care practices (Giezendanner et al., 2018). Barriers such as lack of availability, expertise, and logistics prevent physicians from using such cognitive screening tests (Koch et al., 2010; Sabbagh et al., 2020).

In Switzerland, PCP often use paper‐and‐pencil tests such as the mini‐mental state examination (MMSE) (Folstein et al., 1975) and the clock drawing test to screen patients for cognitive deficits, most of which are not sensitive enough to detect patients with early neurocognitive disorders (Bally et al., 2021). The Association of Swiss Memory Clinics recommends PCP to use the Montreal Cognitive Assessment (MoCA) (Nasreddine et al., 2005) as a cognitive screening tool in clinical practice because it is more sensitive and specific to early changes in cognition than other paper‐and‐pencil‐based tests (Bürge et al., 2018; Pinto et al., 2019). However, despite its good psychometric characteristics to detect mild neurocognitive disorder, particularly due to Alzheimer's disease (AD) (Pinto et al., 2019; Tsoi et al., 2015), the MoCA has several disadvantages for early diagnosis in primary care settings including lack of assessment of non‐cognitive symptoms that belong to the first symptoms of atypical non‐AD neurodegenerative disorders such as frontotemporal dementia, informant ratings, functional decline, as well as automatic interpretative feedback for PCP to guide care. To overcome some of these problems, researchers have recently developed a tablet‐based screening tool that includes a very brief, 10‐min cognitive assessment (brain health assessment, BHA) of four key cognitive domains (memory, executive functioning, visuospatial functioning, language), and an optional informant survey (brain health survey, BHS) (Possin et al., 2018). This validation study conducted in a sample of highly educated, White, and English‐speaking patients in the United States showed that the tablet‐based cognitive assessment (BHA) alone, as well as in conjunction with the informant survey, shows excellent combined sensitivity and specificity to typical and atypical presentations of mild neurocognitive disorder and dementia, outperforming the widely used MoCA (Possin et al., 2018). In addition, a longitudinal follow‐up study demonstrated that the instrument is also sensitive to changes over time, even to very subtle changes in healthy individuals with positive amyloid status (Tsoy et al., 2021). Validation studies in different Western and developing countries are ongoing, and the first study completed in a Hispanic sample in Cuba also showed that the BHA remains sensitive and specific to detect early symptoms of patients with neurodegenerative disorders in that population (del Alamo et al., 2003). Our study presents the first validation study conducted in a German‐speaking sample that has the potential to provide further cross‐cultural evidence for the usefulness of the tablet‐based screening tool for early diagnosis and to track cognitive changes in primary care practices.

In this multi‐site study, we translated the BHA and BHS to standard high German and investigated whether the German version can also distinguish patients with mild and major neurocognitive disorder from healthy controls at high sensitivity and specificity. Based on the results obtained in the English‐ and Spanish‐speaking samples, we expected that the German version would accurately indicate early clinical symptoms of individuals with neurocognitive disorders.

## 2 METHODS

### 2.1 Participants

This cross‐sectional, observational study was conducted at three sites in Switzerland: the Memory Clinic of the Kantonsspital St. Gallen, the Memory Clinic of the Geriatrische Klinik St. Gallen, and the Memory Clinic Universitäre Altersmedizin Felix Platter Spital Basel. We included 29 patients fulfilling the Diagnostic and Statistical Manual of Mental Disorders (DSM)‐5 (American Psychiatric Association, 2013) criteria for mild neurocognitive disorder which include evidence of modest cognitive decline from a previous level of performance in one or more cognitive domains evidenced by both a concern of the individual, an informant, or the clinician, and a modest impairment in cognitive performance. The diagnostic criteria require that the cognitive deficits do not interfere with the capacity for independence in everyday activities. In addition, we enrolled 38 patients with major neurocognitive disorder based on the DSM‐5 (American Psychiatric Association, 2013) criteria. These patients were required to show evidence of cognitive decline from a previous level of performance in one or more cognitive domains based on both concern of the individual, an informant, or the clinician, and a substantial impairment in cognitive performance. The cognitive symptoms associated with major neurocognitive disorder interfere with patients’ independence in instrumental activities of daily living such as paying bills or managing medications. Furthermore, patients with major neurocognitive disorder fulfilled the research criteria for one of the following clinical syndromes: AD clinical syndrome (Bennett et al., 2018) (*n* = 19), vascular cognitive disorder (Sachdev et al., 2014) (*n* = 1), mixed pathology (AD and vascular; *n* = 11), behavioral variant frontotemporal dementia ( *n* = 3) (Rascovsky et al., 2011), semantic variant primary progressive aphasia (*n* = 2) (Gorno‐Tempini et al., 2011), nonfluent variant primary progressive aphasia (*n* = 1) (Gorno‐Tempini et al., 2011), or logopenic variant primary progressive aphasia (*n* = 1) (Gorno‐Tempini et al., 2011). Exclusion criteria for all patients included other neurological disorders (e.g., stroke and traumatic brain injury), delirium, severe psychiatric illnesses (e.g., schizophrenia), alcohol and substance abuse, being unable to give informed consent due to any reason (e.g., severe cognitive impairment), and MoCA score ≤10/30.

Patients were recruited either at the Memory Clinic of the Kantonsspital St. Gallen (*n* = 55) or at the Memory Clinic of the Geriatrische Klinik St. Gallen (*n* = 12). Each patient underwent a comprehensive diagnostic evaluation, including a clinical interview, neuropsychological and neurological evaluation, and brain magnetic resonance imaging. A subset of patients (60 out of 67) received a lumbar puncture to identify amyloid pathology. The final diagnoses were made by a multidisciplinary team of neurologists, neuropsychologists, and neuroimaging experts. The team also evaluated patients’ capacity to provide written informed consent.

In addition to patients with mild and major neurocognitive disorder, we included a sample of 50 healthy controls recruited from a database of 3000 healthy individuals at the Memory Clinic Basel. To confirm that these individuals were cognitively healthy (MoCA score ≥26), the German version of the MoCA (Nasreddine et al., 2005) was performed. Exclusion criteria of the healthy control group were assessed using a medical questionnaire developed at the Memory Clinic Basel, which was used to rule out subjective cognitive decline (Jessen, 2014) and a history of neurological disorders, psychiatric disorders, as well as alcohol and substance abuse.

For each patient and healthy control subject, one relative (e.g., spouse, parent, sibling) or close friend who had known the participant for 5 or more years and was in regular contact with the subject was enrolled in the study. Informants with neurocognitive or acute psychiatric disorders were excluded. This group of relatives was included to collect informant‐based ratings on the BHS. The study was reviewed and approved by the Swiss Ethics Committees Ostschweiz (EKOS) and Nord‐ und Zentralschweiz (EKNZ). Written informed consent was obtained from all participants and their study partners.

### 2.2 Behavioral measures

The German BHA, BHS, and MoCA were administered to all patients and healthy control subjects. The BHA and BHS are programmed in the TabCAT software developed at the University of California, San Francisco (UCSF) (https://memory.ucsf.edu/tabcat), available on the Apple App Store. The BHA, the main outcome measure of this study, is a 10‐min tablet‐based cognitive assessment comprising four key domains (memory, executive functioning, visuospatial functioning, and language). The Favorites task measures episodic memory and requires participants to learn associations (four faces each paired with a food and an animal) in two learning trials. After each trial, each face reappears on the screen, and participants have to recall the food and animal associated with each face. The task also includes a 10‐min delayed recall and a recognition trial. The Match task is a measure of executive functioning/processing speed that requires participants to assign symbols to numbers as quickly as possible within 2 min. In the Line Orientation task, a measure of visuospatial functioning, participants have to decide which of two target lines is parallel to a reference line. The difficulty of the Line Orientation task is adaptive, that is, individually adjusted during the task to precisely fit the performance of the participant, and the final score represents the threshold of the number of degrees of difference a participant can reliably distinguish, with lower scores reflecting more fine‐grained discrimination. Finally, the Animal Fluency task was used to assess language skills. Participants were asked to name as many animals as possible within 1 min (Libon et al., 2009). The number of correct responses, repetitions, and rule breaks was documented. For the Favorites, Match, and Animal Fluency tasks, a higher score represents better performance.

The BHS is an optional informant survey asking 24 questions about typical cognitive, behavioral, and motor symptoms of neurocognitive disorders, functional impairment, and rapidity of decline. Informants were asked to assess changes in cognition, behavior, and motor functioning as well as patients’ functional level within the past 5 years. They had to rate each question on a Likert scale, which either consisted of the three response options “yes,” “no,” and “don't know,” or the four response options “no change,” “questionably worse,” “a little worse,” and “much worse.”

To compare the sensitivity to mild neurocognitive disorder between the tablet‐based BHA/BHS and existing paper‐and‐pencil cognitive screening tests, the German version of the MoCA (Bartusch & Zipper, 2004) was administered to all participants. The MoCA screens six cognitive domains, including executive and visuospatial functioning, attention, language, memory, and orientation for time and place. The global score ranges between 0 and 30, and a cut‐off score ≥26 was considered cognitively healthy in the original work of Nasreddine et al. (2005).

### 2.3 Translations and statistical analyses

The first step of this study was to translate the tablet‐based BHA and BHS to standard high German. Forward and backward translations were performed by two translators who were proficient in German and English. All statistical analyses were performed using Statistical Analysis Software (SAS) version 9.4. Group differences in demographic and clinical variables were examined using linear modeling (PROC GLM). Group differences in age, sex, and education (which included elementary and high school, as well as university/academic degrees) were analyzed using Tukey post hoc tests. Dunnett‐Hsu post hoc tests were used to compare mean least‐square scores on the BHA, BHS, and MoCA between each patient group and the control group. According to Shapiro–Wilk tests, the distribution in each variable within groups was not normally distributed. Thus, to ensure the robustness of our predictions, we used logistic regression analysis instead of discriminant function analysis to investigate whether the tablet‐based BHA/BHS was able to distinguish (1) patients with mild neurocognitive disorder from healthy controls and (2) patients with major neurocognitive disorder from healthy controls. In a second set of analyses, based on the results of the lumbar puncture, we divided the entire group of patients with mild and major neurocognitive disorder into subgroups of amyloid positive (amyloid+) and amyloid negative (amyloid−) individuals, and reperformed the logistic regression analyses described above. We performed these analyses across patients with mild and major neurocognitive disorder for whom amyloid status was available (*n* = 60) because our patient subgroups were underpowered to perform these analyses separately for patients with mild and major neurocognitive disorder (see Table 1). Similar to the approach used in Possin et al. (2018), we first converted the raw scores of each BHA task to *z*‐scores using a regression‐based approach adjusting for age, sex, and education based on the healthy control sample. Regression‐based norms have higher prediction accuracy compared to traditional norming approaches, particularly for small samples (Van der Elst et al., 2011). Thus, for each BHA task, we performed multiple linear regression analysis in the healthy control sample, including the demographic variables (age, sex, and education) as predictors in each model. For each patient, we then calculated demographically adjusted *z*‐scores for each of the four BHA tasks using the formula *z* = (*Y* − *Y’*)/RSE, where *Y* is the observed raw score, *Y’* is the predicted score derived from the regression model, and RSE is the residual standard error of the regression equation. Because higher scores on the Line Orientation task reflected poorer performance, the *z*‐score calculations for this task were reversed. To compare the sensitivity and specificity of the tablet‐based assessment and the MoCA, we included the four BHA *z*‐scores, the BHS sum score, and the MoCA sum score in our logistic regression models.

**TABLE 1:** Demographic and clinical characteristics of the study sample.

	Healthy controls	Mild neurocognitive disorder	Major neurocognitive disorder	*p*‐value
Total *n*	50	29	38	–
Number of participants with missing BHS^a^ data	3	6	6	–
Number of patients with amyloid status, +/−	—	11/11	29/9	–
Age, *M* (SD)	71.84 ± 7.57	73.21 ± 8.94	74.42 ± 7.47	.316
Sex, female	56%	58.6%	52.6%	.884
Education, *M* (SD)	14.28 ± 2.67	13.34 ± 3.09	13.05 ± 2.79	.107
BHA favorites, *M* (SD)	16.72 ± 4.87	7.31 ± 5.37^*^	4.92 ± 5.68^*^	<.0001
BHA match, *M* (SD)	46.24 ± 6.03	34.97 ± 9.84^*^	30.53 ± 11.10^*^	<.0001
BHA line orientation, *M* (SD)	5.42 ± 2.59	6.92 ± 4.63	7.86 ± 4.82^*^	.016
BHA animal fluency, *M* (SD)	22.86 ± 4.92	17.86 ± 4.63^*^	13.74 ± 5.47^*^	<.0001
BHS, *M* (SD)	0.02 ± 0.10	0.91 ± 1.14^*^	1.47 ± 1.54^*^	<.0001
MoCA total score, *M* (SD)	26.78 ± 0.20	22.55 ± 3.38^*^	19.74 ± 3.81^*^	<.0001

Abbreviations: BHA, brain health assessment; BHS, brain health survey; MoCA, Montreal Cognitive Assessment.

^a^ BHS data were only available for a subset of healthy controls (47 out of 50) and patients with mild (23 out of 29) and major (32 out of 38) neurocognitive disorders because some informants were not willing or cognitively incapable to fill out the questionnaire. Group differences in age, sex, and education were analyzed using Tukey post hoc tests. Dunnett‐Hsu post hoc tests were used to compare mean least‐square scores on the BHA, BHS, and MoCA between each patient group and the control group.

* Group differs from healthy control group at *p* < .05.

Because not all informants were cognitively able or willing to fill out the BHS, the total rate of missing values across the sample of patients (mild neurocognitive disorder, *n* = 6; major neurocognitive disorder, *n* = 6) and healthy controls (*n* = 3) was 13%. There were no missing data for any of the other variables. To include all participants in our statistical analyses, we applied multiple imputation analysis in SAS (PROC MI) to estimate the missing values, using the fully conditional specification method and 13 imputation cycles (White et al., 2011).

## 3 RESULTS

### 3.1 Demographic and clinical characteristics

We attempted to match the two patient groups and the healthy control group as closely as possible and selected the healthy control participants based on each patient's demographics. In line with this approach, the three subgroups did not statistically differ with regard to age, sex, or education (see Table 1). In addition, and consistent with our expectations, the two patient groups had significantly lower scores on the Favorites, Match, and Animal Fluency tasks of the BHA, as well as on the MoCA total score (Table 1). The threshold score of the Line Orientation task was significantly higher, that is, worse (*p* < .05), in patients with major neurocognitive disorder (M ± SD: 7.86 ± 4.82) compared to healthy controls (5.42 ± 2.59). By contrast, the average threshold score of patients with mild neurocognitive disorder (6.92 ± 4.63) did not significantly differ from the healthy control group (5.42 ± 2.59).

### 3.2 Characteristics of the tablet‐based assessment for differential diagnosis

**FIGURE 1:**
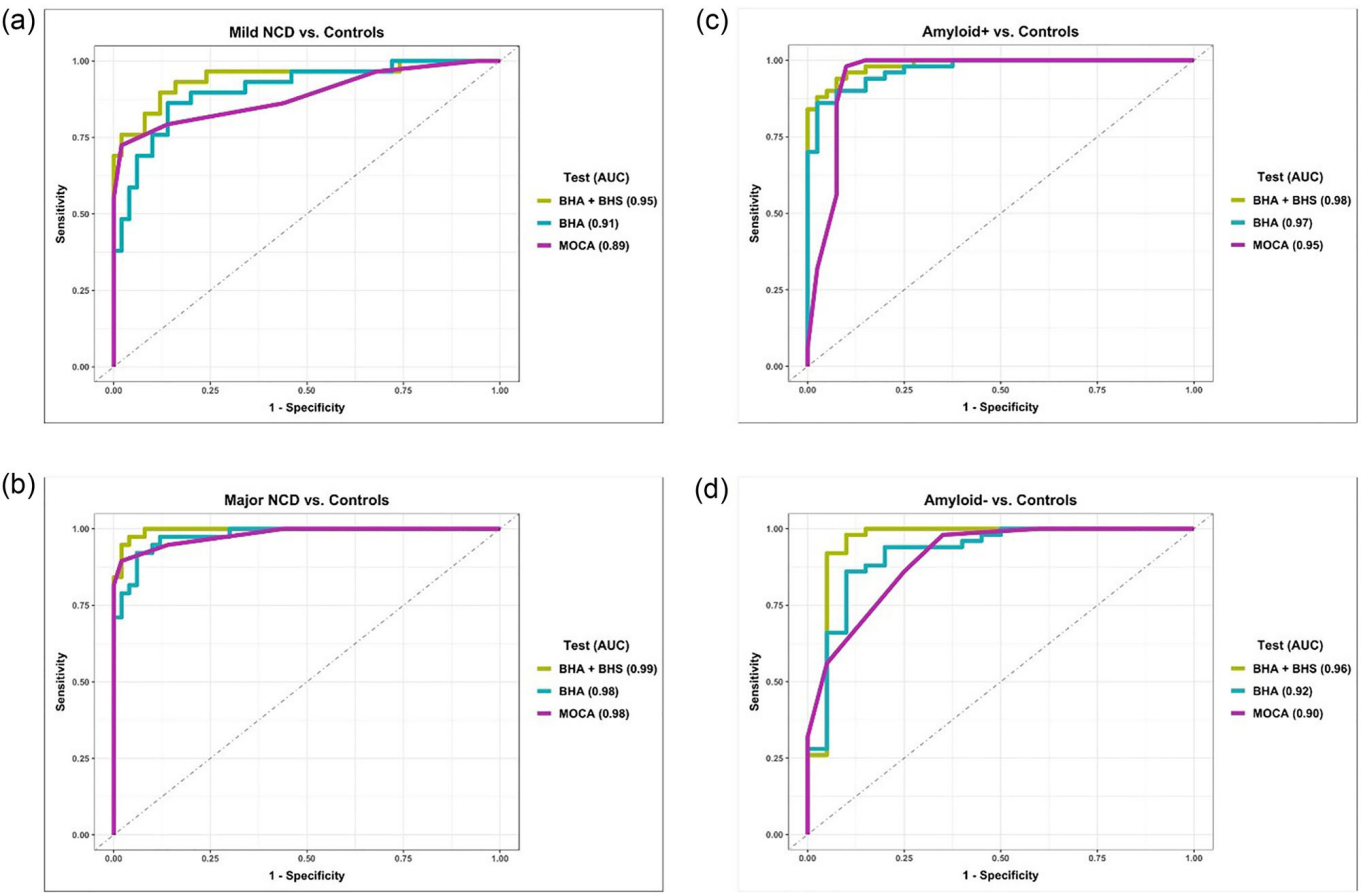
Area under the ROC curves (AUCs) of the complete instrument (BHA + BHS), the cognitive BHA alone, and the Montreal Cognitive Assessment (MoCA) shown for different group comparisons: (a) patients with mild neurocognitive disorder (NCD) versus cognitively healthy controls; (b) patients with major neurocognitive disorder versus healthy controls; (c) patients with positive amyloid (amyloid+) status versus healthy controls; (d) patients with negative amyloid (amyloid−) status versus healthy controls. BHA, brain health assessment; BHS, brain health survey.

To determine how well the German version of the tablet‐based assessment can distinguish between patients and controls, we performed logistic regression analyses and calculated area under the ROC curves (AUCs) for the complete instrument (BHA + BHS), the cognitive BHA alone, and the MoCA total score. For both the distinction of (1) patients with mild neurocognitive disorder versus healthy controls and (2) patients with major neurocognitive disorder versus healthy controls, the complete instrument (BHA + BHS) had the highest AUC when compared with the cognitive BHA alone or the MoCA alone (Table 2 and Figure 1a,b). As shown in Table 2, the AUCs of the three measures, which were all excellent, were comparable for differential diagnosis of patients with major neurocognitive disorder and healthy controls, ranging between 0.98 and 0.99. While the AUCs distinguishing patients with mild neurocognitive disorder from healthy controls were also excellent for the complete instrument (0.95), the AUC for the MoCA was good (0.89).

**TABLE 2:** Area under the ROC curve (AUC) and sensitivity (SEN) for different measures and group comparisons.

	Mild NCD/controls		Major NCD/controls		NCD amyloid+/controls		NCD amyloid−/controls	
	AUC	SEN	AUC	SEN	AUC	SEN	AUC	SEN
BHA + BHS	0.95	0.88	0.99	0.98	0.97	0.98	0.96	0.95
BHA	0.91	0.86	0.98	0.94	0.97	0.97	0.92	0.90
MoCA	0.89	0.75	0.98	0.98	0.95	0.93	0.90	0.75

*Note*: Sensitivity is provided at a specificity level of 85%.

Abbreviations: BHA, brain health assessment; BHS, brain health survey; MoCA, Montreal Cognitive Assessment; NCD, neurocognitive disorder.

In the second step, we investigated the impact of amyloid status on the accuracy of the three measures to correctly distinguish patients from controls. Thus, we examined the AUCs of the tablet‐based assessment and the MoCA for differentiating patients for whom amyloid status was available (positive: *n* = 40; negative: *n* = 20) from controls (*n* = 50). The AUC of the complete instrument was excellent and high in distinguishing patients with (0.97) and without (0.96) amyloid pathology from the healthy control group (Table 2 and Figure 1c,d). While the MoCA showed similar, slightly lower values, it was more accurate at differentiating patients with (0.95) than without (0.90) amyloid pathology from controls.

## 4 DISCUSSION

To improve early diagnosis of patients with progressive neurodegenerative disorders, efficient and accurate cognitive screens are needed for PCP across the world (Borson et al., 2013). Two previous studies have shown that the English and Spanish versions of the tablet‐based UCSF BHA/BHS are more accurate at detecting early symptoms of patients with mild neurocognitive disorder than existing paper‐and‐pencil‐based tests like the MMSE and MoCA. In the present validation study performed at three memory clinics in Switzerland, we confirm these previous cross‐cultural findings from other high‐ and middle‐income countries. We confirmed that the German versions of both the tablet‐based BHA/BHS and the MoCA accurately detected major neurocognitive disorder; however, the complete instrument (BHA + BHS) was more sensitive to (1) mild neurocognitive disorder and (2) neurocognitive disorder due to non‐AD pathology than the cognitive BHA alone or the MoCA. This study suggests that using the standard high German version of the BHA/BHS instrument in primary care practices may result in a higher number of early and accurate diagnoses of typical and atypical presentations of neurodegenerative diseases and of referrals to dementia specialist centers. The usability of this new German version of the tablet‐based BHA/BHS is not limited to Switzerland, but it can be more broadly used in any German‐speaking country in Europe, including Germany and Austria. Our findings are of high importance to both urban and rural areas in these countries where PCP are involved in both the diagnostic decision‐making process and triage of patients to specialists.

The tablet‐based BHA/BHS was previously validated at the clinical, neuroanatomical, and neuropathological level (Possin et al., 2018; Tsoy et al., 2021). The face‐to‐face test includes automated scoring and is easily used even by individuals with cognitive impairment and limited computer experience. The informant‐based BHS consists of 24 questions about the most typical behavioral and motor symptoms seen in patients with frontotemporal lobar degeneration syndromes (Rabinovici & Miller, 2010). Because of the excellent combined sensitivity and specificity of the complete instrument (BHA + BHS) to detect mild and major neurocognitive cognitive disorder regardless of amyloid status, we recommend that, if possible, PCP use both the BHA (10 min administration time with the patient) and the BHS (5 min administration time for the caregiver) in clinical routine. Importantly, both the combined instrument and the cognitive BHA alone had excellent accuracy in distinguishing patients from controls, outperforming the paper‐and‐pencil‐based MoCA. The finding that even the BHA alone accurately detects early neurocognitive disorders with different underlying etiologies is practically relevant because informants cannot always be included in the diagnostic process due to issues such as informant unavailability, diminished cognitive status, and unwillingness to provide information about the patient.

At a specificity level of 85%, the sensitivity of the MoCA to detect mild cognitive impairment was much higher in the Swiss (0.75) sample than the US (0.25) sample. The MoCA was originally developed for the early detection of patients with AD and shows excellent sensitivity and specificity to mild neurocognitive disorder due to underlying AD pathology (Nasreddine et al., 2005). Thus, the discrepancy between the two studies may at least partly be explained by the fact that the Swiss sample consisted of a higher proportion of AD‐related neurocognitive disorders than the US sample.

## 5 LIMITATIONS AND FUTURE DIRECTIONS

The overarching goal of this multi‐site study was to show the validity of the standard high German version of the tablet‐based BHA and BHS for the early diagnosis of neurocognitive disorders. Because the English version of the tool has been thoroughly validated both neuroanatomically and neuropsychologically (Possin et al., 2018), the focus of this study was to validate the instrument on the behavioral level. Due to its brevity, automated scoring, and accuracy for differential diagnosis of early neurodegenerative diseases and cognitively healthy individuals, the German version of the tablet‐based BHA/BHS provides a suitable screening instrument for primary care providers in German‐speaking countries. However, the current study does not provide any information about PCP's perceptions of the usefulness of the instrument or its impact on the diagnostic process in primary care settings. In addition, there are several practice‐level barriers around scheduling and expertise that may prevent physicians from evaluating cognition in routine clinical care (Kaduszkiewicz et al., 2010; Koch et al., 2010; Sabbagh et al., 2020), even in the presence of sensitive instruments such as the BHA and BHS. Therefore, additional implementation work must be done before PCP in Switzerland and other German‐speaking countries can effectively adopt the BHA/BHS instrument, which is a worthwhile endeavor because of its excellent accuracy in detecting early symptoms of neurodegenerative disorders. To directly implement the instrument in primary care practice, health systems must provide guidance around the management of the electronic application, protocols for patient data collection and storage, and provider education around interpretation of results and follow‐up recommendations after cognitive evaluation. These steps would overcome many current health system barriers to implementing efficient and accurate cognitive testing in primary care settings, which in turn would improve an early diagnosis of neurodegenerative disorders and ultimately result in better care of affected patients and families. Finally, future research is warranted to investigate longitudinal models of the German TabCAT‐BHA, including prediction of decline, long‐term stability, and change over time in individuals with early neurodegenerative diseases.

## AUTHOR CONTRIBUTIONS

G.T. and A.F. conceived the study and raised funding. G.T., L.S. and D.K. performed cognitive tests and supported G.T. in the statistical analyses. G.T., L.S., D.K., F.K., T.M., U.K., A.M. and A.F. recruited the patients and collected the clinical data. P.C., K.P., K.R. developed the original tests in english language. G.T. and A.F. wrote the manuscript and all authors contributed to editing and revising the manuscript.

## CONFLICT OF INTEREST STATEMENT

The authors declare no conflicts of interest.

### PEER REVIEW

The peer review history for this article is available at https://publons.com/publon/10.1002/brb3.3329

## Data Availability

Anonymized data will be shared on request from any qualified investigator for the purposes of replicating procedures and results.
