# Correction to “The German version of the tablet‐based UCSF Brain Health Assessment is sensitive to early symptoms of neurodegenerative disorders”

**DOI:** 10.1002/brb3.3394

**Published:** 2024-01-16

Toller, G., Stäger, L., Kumurasamy, D., Callahan, P., Köhn, F., Münzer, T., Kunze, U., Monsch, A. U., Possin, K., Rankin, K. P., & Felbecker, A. (2023). The German version of the tablet‐based UCSF Brain Health Assessment is sensitive to early symptoms of neurodegenerative disorders. *Brain and Behavior*, 13, e3329. https://doi.org/10.1002/brb3.3329

In the published version of the article, the first names and surnames for several authors were inadvertently transposed. The correct authors are: Gianina Toller, Lorena Stäger, Dilaxy Kumurasamy, Patrick Callahan, Florian Köhn, Thomas Münzer, Ursi Kunze, Andreas U. Monsch, Kate Possin, Katherine P. Rankin, and Ansgar Felbecker. The online version of the article has been corrected.

We apologize for this error.

